# Increasing nursing student interest in rural healthcare: lessons from a rural rotation program in Democratic Republic of the Congo

**DOI:** 10.1186/s12960-021-00598-9

**Published:** 2021-04-20

**Authors:** Susan Michaels-Strasser, Paul W. Thurman, Narcisse Mwinkeu Kasongo, Daniel Kapenda, John Ngulefac, Beatrice Lukeni, Serge Matumaini, Lauren Parmley, Rebekah Hughes, Faustin Malele

**Affiliations:** 1 ICAP At Columbia University, 722 West 168th St., 13th Floor, New York, NY 10032 USA; 2 Mailman School of Public Health, Columbia University, 722 West 168th St., 4th Floor, New York, NY 10032 USA; 3 Institut Supérieur Des Techniques Médicales (ISTM) de Lubumbashi, Lubumbashi, Democratic Republic of the Congo; 4 United States Health Resources and Services Administration, 5600 Fishers Lane, , Rockville, MD 20852 USA; 5 ICAP At Columbia University, Lubumbashi, Democratic Republic of the Congo; 6 ICAP At Columbia University, Kinshasa, Democratic Republic of the Congo

**Keywords:** Rural healthcare, Nursing, Rural rotations, Recruitment

## Abstract

**Background:**

Many challenges exist in providing equitable access to rural healthcare in the Democratic Republic of the Congo (DRC). WHO recommends student exposure to rural clinical rotations to promote interest in rural healthcare. Challenges to rural engagement include lack of adequate infrastructure and staff to lead rural education. This case report highlights key steps in developing a rural rotation program for DRC nursing students.

Case presentation

To implement a rural rotation (RR) program, ICAP at Columbia University (ICAP) consulted with students, the Ministries of Health (MoH) and Education (MoE), and nursing schools to pilot and expand a rural rotation program. Nursing schools agreed to place students in rural clinics and communities. Key stakeholders collaborated to assess and select rural sites based on availability of nursing mentors, educational resources, security, accessibility, and patient volume. To support this, 85 preceptors from 55 target schools and 30 rural health facilities were trained of which 30 were selected to be “master trainers”. These master trainers led the remaining 55 preceptors implementing the rural rotation program. We worked with rural facilities to engage community leaders and secure accommodation for students.

A total of 583 students from five Lubumbashi schools and two rural schools outside Kinshasa participated across 16 rural sites (298 students in 2018–2019 school year and 285 in 2019–2020). Feedback from 274 students and 25 preceptors and nursing school leaders was positive with many students actively seeking rural assignments upon graduation. For example, 97% agreed or strongly agreed that their RR programs had strengthened their educational experience. Key challenges, however, were long-term financial support (35%) for rural rotations, adequate student housing (30%) and advocacy for expanding the rural workforce.

**Conclusions:**

With nearly 600 participants, this project showed that a RR program is feasible and acceptable in resource-limited settings yet availability of ample student accommodation and increasing availability of rural jobs remain health system challenges. Using a multipronged approach to rural health investment as outlined by WHO over two decades ago remains essential. Attracting future nurses to rural health is necessary but not sufficient to achieve equitable health workforce distribution.

## Background

This case study presents a novel rural rotation (RR) program in rural Haut Katanga province of the Democratic Republic of the Congo (DRC). Health systems continue to grapple with how to ensure a robust rural health workforce despite long-standing WHO recommendations on rural education, regulation, financing, and support as key measures to improve health equity [1]. In DRC, where most people reside in rural areas, supporting human resources for health (HRH) interest in rural practice is central to health system strengthening and providing healthcare for all [2]. Access to care needs in Haut Katanga are compounded by a higher than national average HIV prevalence and larger rural population. We describe the rationale, steps taken, and methods used to implement the RR program for nursing students in Haut Katanga, a province heavily impacted by HIV and AIDS. The results of two academic years as well as the challenges faced, lessons learned, and persistent health system constraints are provided.

### DRC context and challenges

DRC, a country with a historically high burden of disease, political instability and low health system use, gained independence in 1997 and has received United States President’s Emergency Plan for AIDS Relief (PEPFAR) funding since 2003 [3, 4]. DRC is a country of over 2 million square kilometers that borders seven sub-Saharan African countries. Decades of violent conflict and instability have taken a devastating toll on the country’s economy, human resources, and infrastructure, and coupled with a largely rural population, present several challenges to providing equitable access to healthcare [2]. The health workforce, of which 45% are nurses, is concentrated in the capital Kinshasa and provincial urban areas [5].

The DRC health system is divided into three major levels including the central level led by the Ministry of Health, the intermediate level—including provincial health hospitals and oversight of programs at the level of health zones. The intermediate level, while maintaining a degree of decentralized control in health service inspection and implementation remains beholden to national policies and directives. Most preventive and curative care is provided through over 500 health zones, 300 general reference hospitals and over 8000 health centers. Health centers are staffed by five-member team led by an advanced level (A1) and secondary level (A 2) trained nurse. A laboratorian, receptionist and logistics/maintenance staff member round out the rural team.

Maldistribution of health workers continues to challenge achievement of universal health coverage and the Sustainable Development Goals (SDGs). Improving access to care is essential to achievement of the SDGs, particularly SDG 3 (good health and wellbeing) and 10 (reduced inequalities) United Nations [6]. Nurses, as primary care providers, are central to improving access to care and reducing inequity. Although nurses are the largest health workforce globally and, in the DRC, recruitment and retention of nurses into rural areas remains a challenge. This challenge persists despite the existence of policies to recruit and retain rural health workers [6–8].

The DRC health system is also tasked to respond to a generalized HIV epidemic with an estimated prevalence of 1.2% among adults aged 15 to 49. An estimated 404,894 people are living with HIV, and around 10,535 people die from AIDS-related conditions each year [9]. The HIV epidemic has drastically impacted two of DRC’s most populous provinces, with general population prevalence estimates of 1.6% in Kinshasa and 2.6% in Haut Katanga [10].

### Support to HIV service delivery scale-up in Haut Katanga

ICAP at Columbia University (ICAP) initiated a comprehensive program of support for HIV care and treatment (C&T) services in DRC in 2010. Since then, ICAP has worked hand-in-hand with the Programme National de Lutte contre le SIDA (PNLS) to expand availability, quality, and uptake of adult and pediatric HIV care and treatment in DRC with an emphasis on expanding prevention of mother-to-child transmission (PMTCT) activities, building laboratory networks for disease monitoring, integration of HIV and tuberculosis (TB) services, improvement of infrastructure, prevention among key populations (KP) in Kinshasa and Haut Katanga provinces. The program has expanded from 10 sites in 2010 to 240 public and private hospitals, health centers, and TB clinics as of September 2014. ICAP currently supports the health ministry in 199 sites.

### Support to nurse strengthening in Haut Katanga

With PEPFAR funding through the United States Health Resources and Services Agency (HRSA), in 2017, ICAP was awarded the Resilient and Responsive Health Systems (RRHS) project to continue strengthening HRH in DRC. Using the World Health Organization (WHO) conceptual framework for HRH development, the first 2 years of the RRHS project built on extensive HRH capacity-building and infrastructure improvements for student nurses and midwives through the HRSA-funded Nursing Education Partnership Initiative (NEPI) and Global Nursing Capacity Building Program (GNCBP) [11, 12]. NEPI focused on nurses’ readiness for clinical practice through curricula reform and development of innovative pedagogy including use of skills labs and simulation-based training. Over the past 2 years, HRSA and ICAP have leveraged these pre-service strengthening efforts to shift focus to in-service capacity-building and addressing wider HRH limitations affecting epidemic control specifically in largely rural Haut Katanga province.

Given the volume of people living in rural areas and HIV service needs therein, a collaborative effort between the MoH, Ministry of Education (MoE), ICAP and HRSA has yielded a RR program to increase nursing student and community health worker (CHW) exposure to rural health needs as well as rural clinical practice and community engagement prior to graduation, entry to practice, and employment. Additional strategies, not presented here, included the development of a telementoring program to bridge rural health centers with urban specialists and trainers as well as assistance with national registration of nurses.

### The need to expose and engage nursing students in rural healthcare

An increase in the use of telemedicine globally has expanded access to quality healthcare services regardless of a patient’s proximity to physical medical care/clinic services [13, 14], diminishing healthcare disparities in rural settings. However, many countries, including those in sub-Saharan Africa, still struggle with gaps in healthcare access for rural populations. Common themes seen among the extant literature include lack of funds to enhance rural healthcare, lack of medical professionals among rural populations, and lack of technology available for use in rural areas [15]. Current scholarship identifies various issues faced in attempting to close these gaps between rural and urban health settings and describes various interventions that have been used globally, in sub-Saharan Africa, and more specifically in DRC. In particular, the evolving role of nurses in supporting—and advancing—rural healthcare in such contexts is explored in-depth as is the need to actively recruit nurses to areas by demonstrating the career advantages that such experiences and expertise bring. This, exposure, in turn, will be valued more by student nurses and will, as a result, increase their willingness/interest in rural placement [16].

An interesting healthcare paradox exists in rural settings, compared to urban ones, no matter where one looks in the world: rural communities in which some of the most numerous, complex, and diverse healthcare needs exist are precisely those served least by HRH. Despite the prevalence of unmet rural healthcare needs, healthcare providers tend to remain in wealthier, better resourced, urban areas [17]. A retrospective review of 174 nations and the rural “deficits” in health coverage showed that four basic inequities exist when comparing rural versus urban healthcare: lack of rights to healthcare (i.e., fewer entitlement programs in rural areas), shortages of rural HRH, unequal funding for rural health protection (and for preventative healthcare), and high out-of-pocket costs for rural populations forced to pay for their own health services [18] These deficits were seen in very poor rural areas throughout regions of Africa including countries such as Zambia, Nigeria, South Africa and Kenya.

These inequities, alone, however, are not the only reasons for higher incidence and prevalence of some infectious diseases (e.g., malaria), malnutrition, and less preventative care in rural areas. Social determinants of health also come into play including access to education, food and social support [19]. Given social and economic inequities, few rural areas send students to medical and nursing schools; thus, fewer return to serve these areas [15]. Yet where a medical professional student comes from is highly correlated with where that student will practice upon graduation [20]. Attempts to improve recruitment from rural areas—via incentives and increased access to education—have resulted in mixed, or at best only short-term results and improvements [21]. In addition, poor road networks and physical distances from patients to care centers—as well as the social factors and pressures in some rural communities—also prevent patients from seeking health care early or often. Social exclusion from urban areas, not to mention the social pressures to seek guidance from local/rural “healers,” are strong forces that may keep patients from seeking life-saving care [22].

Rural determinants of health result in relatively lower health indicators and indices; for example: greater numbers of stillbirths and higher infant mortality in Central Africa [23], greater child malnutrition and inequitable food security among families across most low-income countries [24], and larger family sizes [25]. If rural determinants of health are not addressed proactively, poor rural health outcomes can pose serious health risks to urban areas—and their healthcare systems and populations, especially after conflict and/or national disasters when (rural) healthcare services are often depleted, and rural urban migration ensues [26]. For example, when an Ebola patient traveled from a small Liberian town to an urban center to seek better care during civil unrest and to escape pressures to utilize local healers, this patient’s travels resulted in several downstream infections closer to an urban center [27].

Failure to address rural healthcare staffing and care provision leads to underreporting of diseases, such as Buruli ulcer in DRC [28], lack of appropriate and basic diagnostic services such as radiology in across sub-Saharan Africa [29], and inadequate communication networks and sharing of best practices from urban to rural settings—from healthcare providers and thought-leaders in DRC [16]. Several interventions have been proposed—including e-health information sharing and application deployment through smartphones in Ghana [30], e-health solutions for rural clinics in South Africa [31], Ghana, Tanzania, and Burkina Faso [32], and improved clinic leadership training for rural healthcare leaders in South Africa [33], yet multipronged HRH development is needed to improve health, avert pandemics and sustain hard won gains in HIV scale-up [12].

In 2019 with support from HRSA, ICAP launched a telementoring program in DRC to help address some of disparities and lack of knowledge transfer from urban to rural settings [34]. While successfully launched and currently being scaled up to, telementoring does not address insufficient supply of health care workers in rural areas able to drive health promotion, disease prevention and treatment. Therefore, a combination approach to strengthening rural health is needed.

Engaging students in rural contexts while increasing their familiarity and understanding of rural health needs allows students to experience the breadth of health services provided in rural areas and opportunities to expand knowledge and skills. Under the mentorship and supervision of experienced rural clinicians, students are exposed to a broader array of healthcare challenges than may be seen in urban settings where care is divided between many units and specialized services. This level of exposure to health issues, symptomatology, and engagement in all aspects of care from health history, point of care laboratory investigations, diagnosis and follow up can rapidly build clinical competence. Students live in and absorb more varied cultural environs and may establish early career leadership and managerial success on a scale more demonstrable than in urban settings where one is “lost in the crowd” of the larger healthcare workforce. Exposing nursing students to the opportunities in rural setting scan create more HRH who are willing to work in rural settings after graduating [35].

## Case presentation

This case study describes the preparation phase and 2 years of implementing a RR program. The preparatory phase including three distinct, sequential yet complementary activities including stakeholder engagement, practicum planning (with nursing schools) and clinic preparation (with health facilities receiving nursing students).

### A. Preparation

#### Stakeholder engagement

During the alignment phase of the project, ICAP/RRHS conducted meetings with the DRC MoH, MoE, Nursing Council, Midwife Association, target schools, students, and students’ parents to raise awareness of the subject by explaining to them the requirements of Education Reform using a competency-based approach:

- Train students to be future health workers on a global scale.
- Prepare them to work in all conditions, even in the remote environments both in hospitals and in rural communities.

Once consensus was reached with key stakeholders on key competency goals, the joint ICAP/RRHS team partnered with nursing schools to support the RR launch.

#### Practicum planning with nursing schools

Once key stakeholders understood the value of building rural practice core competencies in nursing students, getting the nursing schools, themselves, on board was a critical next step. Collaborating to define how the program would be implemented, how progress would be measured, which sites would use, and what safety and security guarantees would be needed were crucial consideration during the planning phase. Once these discussions took place and nursing schools agreed to the RR plan, the team had to identify clinics that were willing to receive nursing students on short-term, rural rotations followed by post-rotation debriefings and continued coursework.

#### Preparing clinics to receive nursing students

Preparing to receive students at different clinic sites took considerable time and energy including site-level assessment, preparation, and education. Sites were assessed for readiness to accommodate students (including students’ logistical and education needs), availability of space in which students could live, learn and practice, availability and quality of preceptors to monitor and evaluate their learning, and broader community acceptance of an influx of student nurses and healthcare provision by nursing students.

**Fig. 1:**
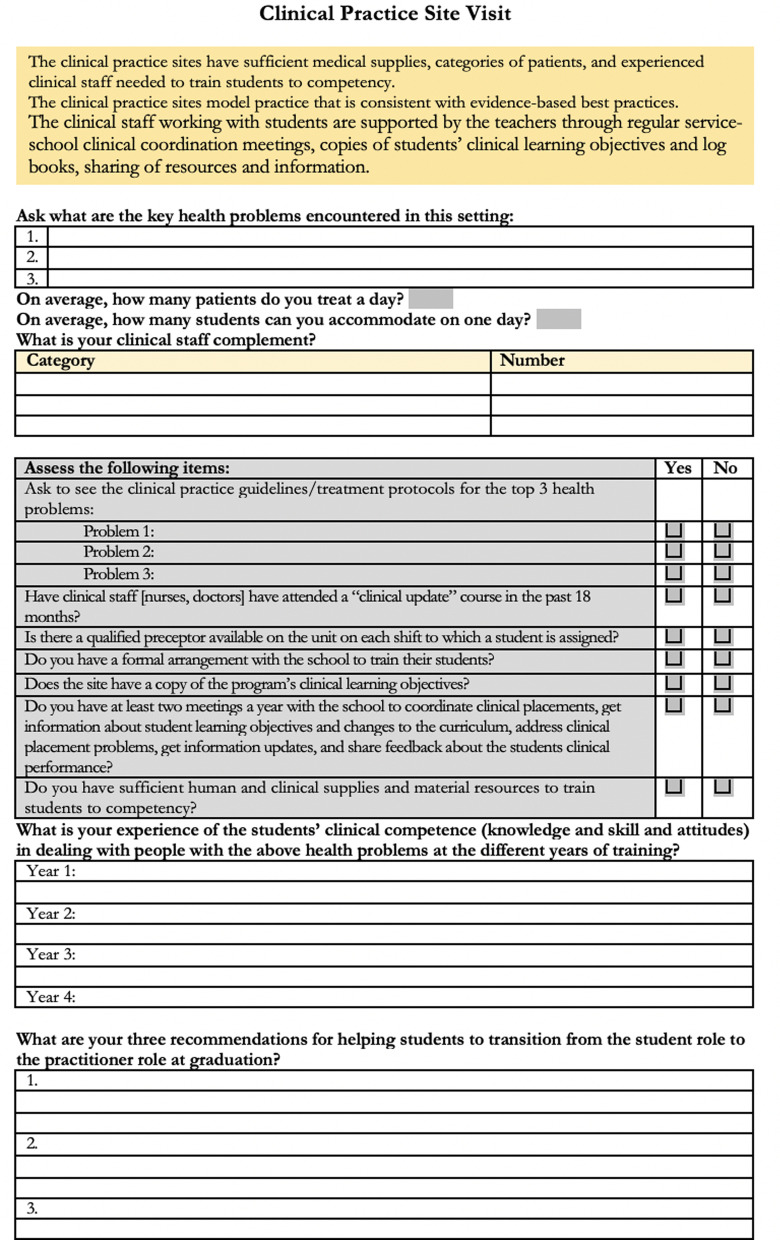
On-site clinical practice site visit assessment tool

**Fig. 2:**
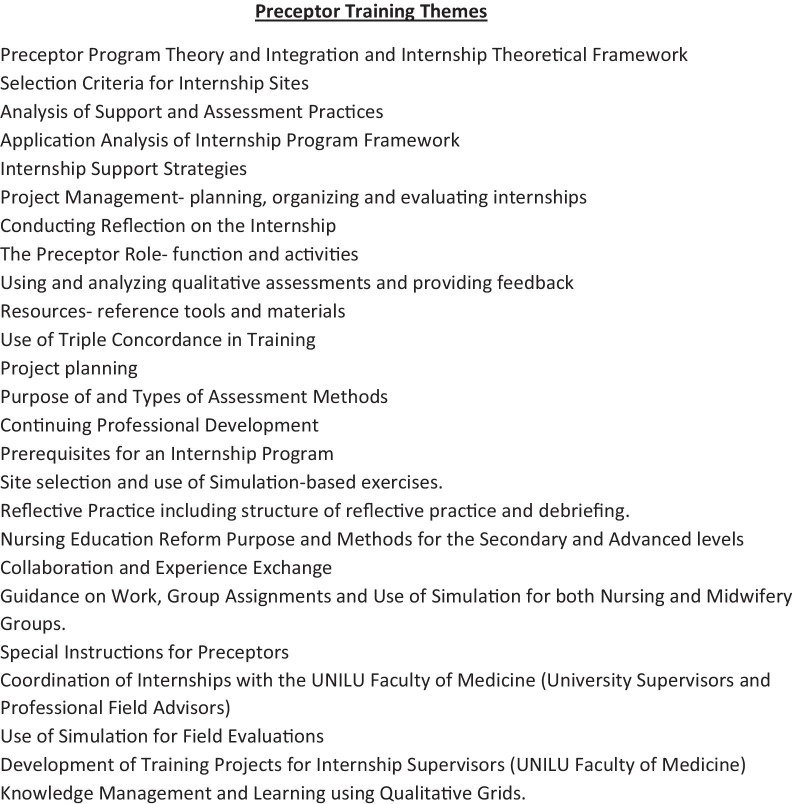
Topics and competencies for preceptor training (in English)

**Fig. 3:**
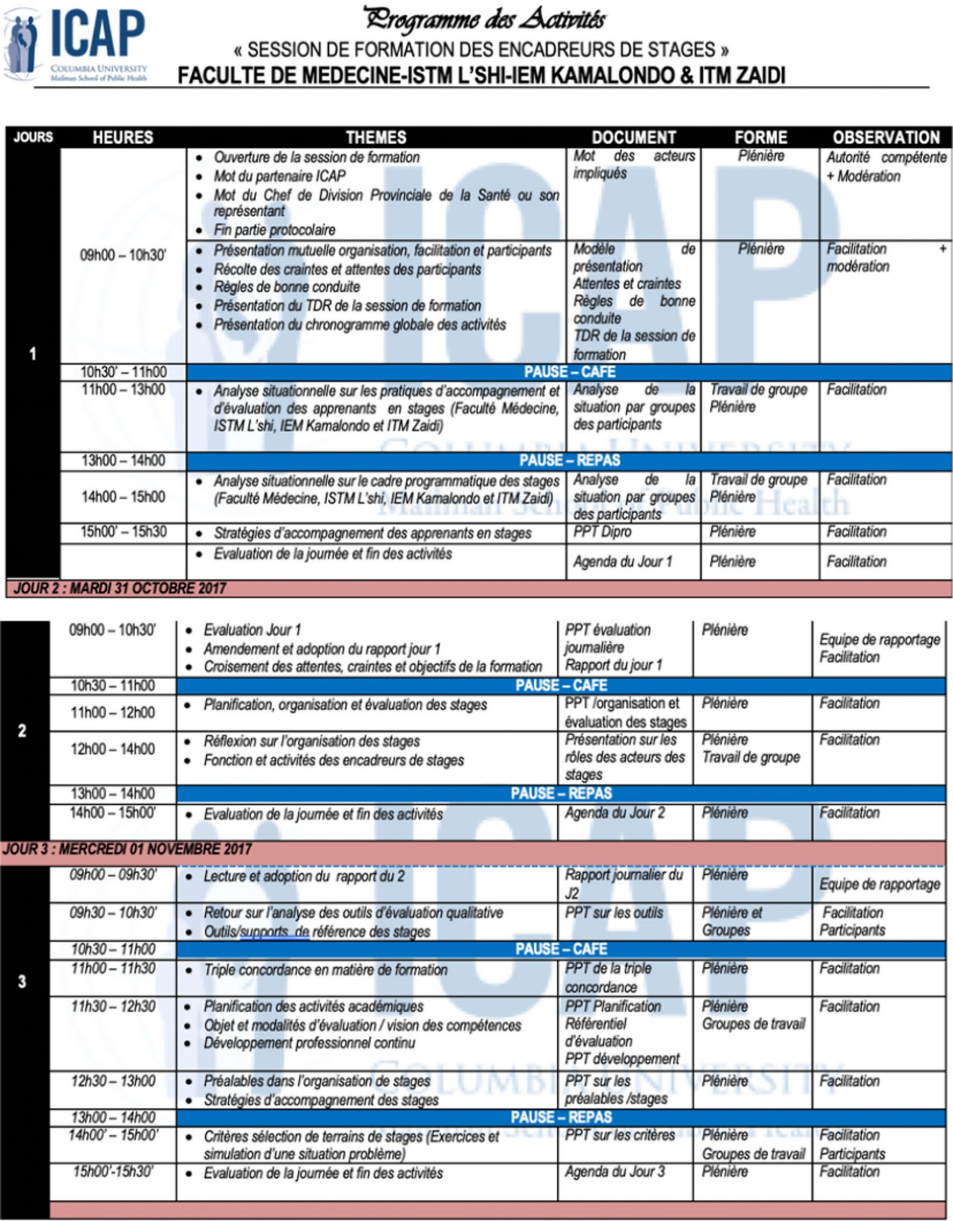

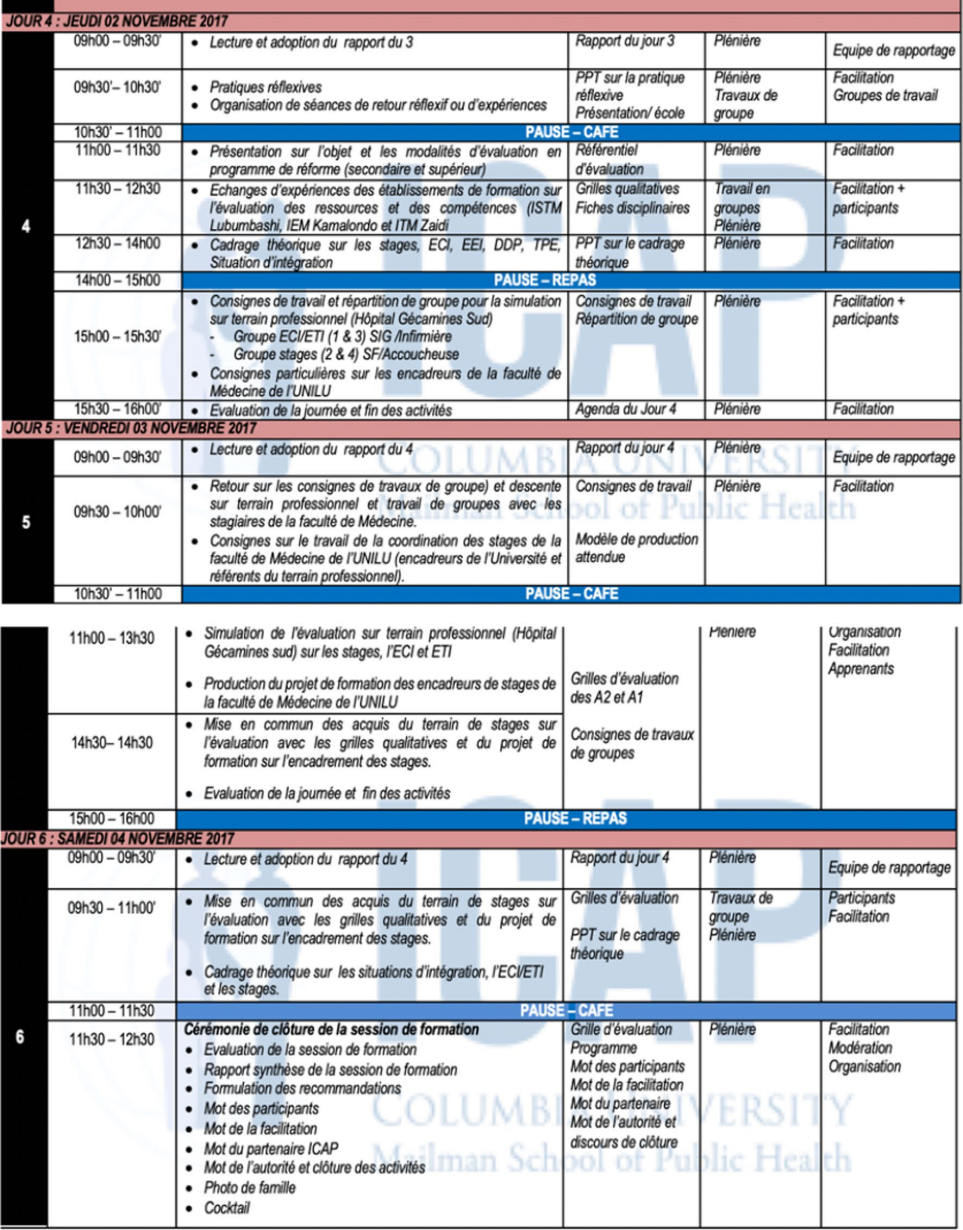
Detailed agenda for preceptor training (in French)

- Mapping of rural sites
  To start, DRC MoH and MoE, working in conjunction with ICAP, identified specific rural sites most likely to be able to handle an influx of students. The ministries informed selected sites and set expectations for the rotational program.
- Assessment of rural sites
  Next, a joint MoH/ICAP team conducted site visits to assess conditions and readiness of the target clinics to receive students. A MOH senior nurse and clinician in charge of health zone technical support, as well as a policy and training manager, and a monitoring and evaluation specialist from ICAP, visited each site and conducted surveys of living and teaching conditions. Site-level assessments focused on four key areas:
- Availability of clinical and teaching materials and equipment (including pathologies)
- Accessibility, security, and accommodation
- Health center volume
- Quality and number of health workers including availability of preceptors.
  A detailed site assessment tool was developed to facilitate site-level collaboration and assessment (see Fig. 1).
- Training of preceptors
  After completing site-level assessments, preceptors from schools and rural facilities were identified for training by the MoH and MoE with ICAP/RRHS technical assistance. Preceptor training focused on how to mentor and train student nurses using a competency framework. To drive efficiency in the training of preceptors, a training of trainers’ approach was used whereby the experts and technical assistants selected a subset of preceptors to complete detailed training. These “master trainers” then trained the remaining preceptors at the various sites. Key topics and competencies covered are presented in Fig. 2, and an example detailed agenda for the multi-day train-the-trainer program can be found in Fig. 3.

Concurrently, MoH, MoE and ICAP continued to align with target clinics to obtain buy-in to the RR program educational aims, objectives and how the program could help to address persistent HRH challenges. Program staff met with community leaders, religious leaders, local authorities, and elders, to explain the program, obtain community feedback and to practically identify accommodations for nursing students. Community engagement was an important step in community acceptance; to preempt/mitigate challenges which could arise from nursing students rotating in and out of their communities.

### Implementation outcomes and challenges

Following assessment of the rural sites, 85 preceptors from 55 target schools and 30 rural health facilities were chosen to be trained as preceptors. Of the total 85 preceptors, 30 individuals completed detailed training with the experts from the MoH and MoE. The 30 individuals were then considered “master trainers” who then trained the remaining 55 preceptors at various sites. As of May 2020, the 30 master trainers have on-boarded an additional 263 preceptors to improve nurse mentorship and instruction during students’ rotations.

All five targeted schools in Lubumbashi participated in the RR program. The DRC urban capital, Kinshasa, was not targeted for RR programs, but two schools supported under NEPI expressed interest and accepted rotating nursing students (Kongo central and rural areas in Kinshasa at the Minkawa Catholic Church health area).

Sixteen sites took part in the RR program. In the 2018–2019 academic year rotations lasted 1 month for secondary level nurses and 3 months for advanced nurses. Participation by nursing schools were broken out as follows:

- ISTM Lubumbashi: 141 nursing students
- IEM Kamalondo: 54
- ITM Immaculée Conception: 65
- ITM Zaidi: 38

In the 2019–2020 academic year, participation, delayed before of COVID-19 has thus far comprised:

- ISTM Lubumbashi (February–July 2020): 87 nursing students
- IEM Kamalondo (February–March 2020): 72
- ITM Immaculée Conception (March 2020): 81
- ITM Zaidi (February–March 2020): 45

Nursing student experienced a wide variety of rural healthcare services including patient care, and community engagement. Students attended to patients during clinic hours and established themselves as valuable community members through outreach (e.g., HIV testing), preventative health advocacy (e.g., importance of improved diets and exercise with local healers and community leaders), and as role models for future nursing students and CHWs.

After completing their RRs, students provided voluntary feedback to inform and improve RRs. 274 students, as well as 25 preceptors and nursing school leaders, provided feedback (see Table 1 for distribution of respondents by site). Eighty percent of students providing feedback were placed during the most recent (2019–2020) academic year, and most students were placed for 1 month (63%) compared to 3- (26%) or rarely, 4-month (11%) placements.

**Table 1:** Distribution of student RR satisfaction survey respondents by site, placement year, and RR duration

Site placement for rural clinical rotation				
Q1	Frequency	Percent	Cumulative frequency	Cumulative peSrcent
Chanfubu General Hosptial	6	2.19	6	2.19
Kabiasha	13	4.74	19	6.93
Kaboka General Hospital	56	20.44	75	27.37
Kaboka Health Center	15	5.47	90	32.85
Kasameno CSR	23	8.39	113	41.24
Kashobwe HGR	20	7.30	133	48.54
Katabe CSR	8	2.92	141	51.46
Kawama Health Center	14	5.11	155	56.57
Kilwa General Hospital	41	14.96	196	71.53
Kisamamba Health Center	4	1.46	200	72.99
Lukonzolwa Health Center	6	2.19	206	75.18
Lumata Health Center	13	4.74	219	79.93
Lwanza Health Center	5	1.82	224	81.75
Mubanga Health Center	9	3.28	233	85.04
Makupa Health Center	13	4.74	246	89.78
Other	28	10.22	274	100.00

Placement year				
Q2	Frequency	Percent	Cumulative frequency	Cumulative percent
2018-2019 Academic Year	54	19.71	54	19.71
2019-2020 Academic Year	220	80.29	274	100.00

Q3	Freqeuncy	Percent	Cumulative frequency	Cumulative percent
1 month	172	62.77	172	62.77
3 months	71	25.91	243	88.69
4 months	31	11.31	274	100.00

Respondents were asked whether they strongly agreed, agreed, disagreed, or strongly disagreed with the following statements:

- I would recommend that my classmates participate in a RR.
- I feel that the RR has strengthened my educational experience.
- I feel better-equipped to provide HIV/AIDS prevention, care, and treatment services because I took part in the RR.

**Fig. 4:**
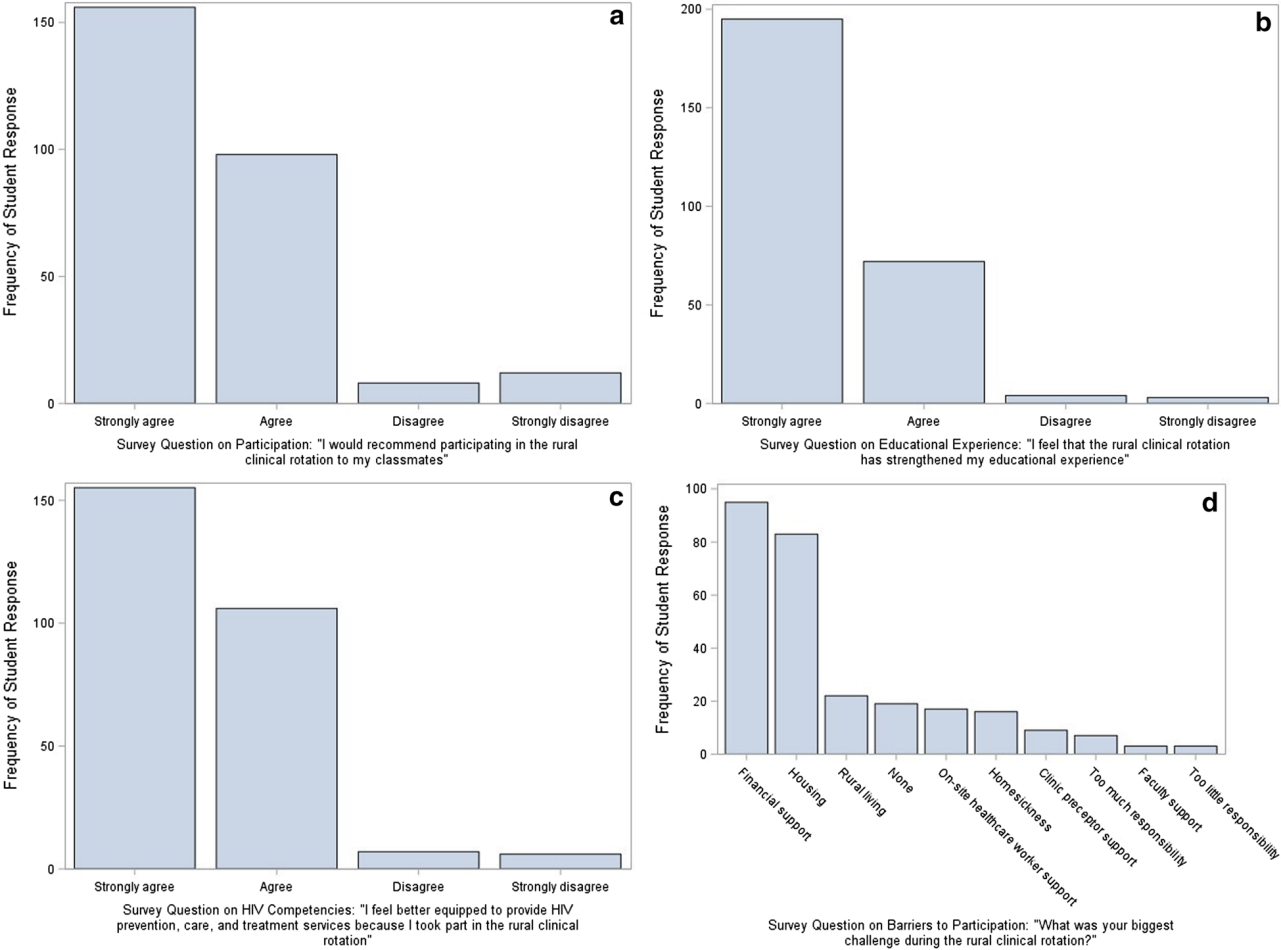
**a** Distribution of RR student responses to the question regarding recommending participation in a RR to classmates. **b** Distribution of RR student responses to the question regarding whether RR participation strengthened educational experience. **c** Distribution of RR student responses to the question regarding whether respondents felt better equipped to provide HIV/AIDS care given RR participation. **d** Distribution of RR student responses to the question regarding biggest challenge during RR

Ninety-three percent of students agreed or strongly agreed that they would recommend RR participation (see Fig. 4a). Ninety-seven percent agreed or strongly agreed that their RR had strengthened their educational experience (see Fig. 4b). Ninety-five percent of students agreed or strongly agreed that they felt better equipped to provide HIV/AIDS prevention, care, and treatment services after RR participation (see Fig. 4c).

When asked about their biggest RR challenges, students most reported financial support (35%), housing (30%), and rural living conditions (3%). Levels of responsibility and preceptor support were also reported by a few respondents (see Fig. 4d). The ICAP team continues to try to correlate RR participation directly with improvements in Monitoring, Evaluation, and Reporting (MER) statistics as reported via PEPFAR “95-95-95” measures.

## Discussion and conclusions

### Rural rotation successes

This 2-year case study demonstrates that a RR program is feasible in low-resource settings. Complementary efforts to register rural nurses and increase access to the latest information and specialist support through telementoring were simultaneously introduced and scaled up. Systematic preparation, stakeholder engagement, site assessment and training of preceptors were critical steps to the program’s early success. Collaboration, a detailed rotational schedule, and developing accommodation for students and preceptors were keys to success.

Student feedback indicates increased opportunities to engage patients and to take part in community-based programs such as home visits, behavior change education, community awareness and health promotion. These activities enhance student exposure and experience in the breadth and depth of primary and acute clinical care. Nursing school leaders and clinic managers reported student engagement to be an asset rather than a burden given the paucity of health workers available in rural settings.

### Rural rotation sustainability

Through donor support, ICAP has supported minor renovations at all 16 sites to improve accommodation conditions and assistance for student and preceptor transportation. In the first year of RR implementation, students were placed at all 16 sites. However, in the second year of placements, only 15 of the 16 sites were staffed due to security issues at one of the sites. The renovations completed on the living quarters for nursing students ensured students would have a secure rural residence. As sustainability is a key goal of this program, ICAP has worked with HRSA, MoH, and MoE to develop a transition and sustainability plan.

Specifically, the programs School Sustainability Plan includes ensuring ongoing maintenance of living accommodations for nurses, continued transportation support, and longitudinal tracking of key educational outcomes to demonstrate program success (including downstream increases in rural nurse placements). By seeing this value from the RR program, continued funding and support has been secured for the foreseeable future. With the onset of COVID-19 and evolving healthcare priorities, rotating nursing students have been uniquely positioned to provide immediate frontline support. As such, DRC MoH and MoE have realized even more value from this program than originally envisioned and have committed to continued funding of RR activities like these in the future.

### Rural rotation challenges

Key challenges to initial launch and ongoing operation of the RR program revolved mostly around accommodation availability and student financial assistance. Not all sites provided accommodations for student nurses, and at those where accommodations were available, spaces were poorly maintained and in need of renovation. Some nursing students also faced financial difficulties including support for transport to rural clinic sites. While the ICAP team was successful in securing some MoH/MoE funding/resources to support students’ needs, these challenges must continue to be addressed to ensure long-term viability of such a program.

### Striving for equity in access to healthcare

This case study has focused on building a nursing RR program recognizing the centrality of nurses to improving access to care. It has shown that a RR program is both acceptable and feasible in remote areas of DRC. Strong support from students, local staff, educators, and health administrators is evident. Broad stakeholder engagement and capacity-building are keys to this success and eventual transition to local ownership and sustainability. Early positive results have been presented using qualitative data and quantitative results to the outcome level. Further study of the essential components as well as short- and longer-term benefits and sustainability of a rural rotation program is encouraged. Further study of the impact of such a program on the recruitment and retention of rural health workers following a rural rotation program as well as the impact of access to health care and health care outcomes is urgently needed. Yet this cannot occur in a vacuum. Using a multipronged approach to drive full implementation of recommended policies and measures to safeguard health workers are required [1, 8]. Identifying and studying key endpoints such as improvements in rural access to care, health outcomes, patient satisfaction, and the professional benefits of rural practice will help strengthen and build on the work presented here. Ongoing outbreaks of Ebola and the current COVID-19 pandemic bring into sharp focus the necessity and urgency to build strong health systems that protect health workers who serve as the frontline of global health security [36].

While the literature clearly shows that exposing students to rural practice is an important strategy to improve interest in and appreciation for rural care [28] and a rural rotation program is now ongoing in Haut Katanga, the recruitment and retention of health workers in rural areas remains a challenge due to comparatively poorer working conditions and lower salaries. In the DRC shortages of staff and the consequent heavy workload for rural nurses and midwives continues to be compounded by the lack of basic supplies such as gloves and medicines [7]. While this case study shows that a rural rotation program for large numbers of nursing students is both acceptable and feasible, using a multipronged approach to rural health investment as outlined by WHO over two decades ago remains essential. Attracting future nurses to rural health is necessary but not sufficient to achieving equitable health workforce distribution.

## Data Availability

Data collected and used for this study are available from the corresponding author upon reasonable request.

## Abbreviations

C&T: Care and treatment
CHW: Community Healthcare Worker
COVID-19: Coronavirus disease 2019
DRC: Democratic Republic of the Congo
GNCBP: Global Nursing Capacity Building Program
HIV/AIDS: Human immunodeficiency virus/acquired immune deficiency syndrome
HRH: Human Resources for Health
HRSA: (United States) Health Resources and Services Administration
ICAP: ICAP at Columbia University
IEM: Institut d'Enseignement Medical (nursing, midwife, technician, and pharmacy school))
IRB: Institutional Review Board
ISTM: Institut Superieur des Techniques Medicales (nursing and medical school)
ITM: Institut des Techniques Medicales (nursing and midwife school)
KP: Key populations
MER: Monitoring, Evaluation, and Reporting statistics (from PEPFAR’s 95-95-95 goals)
MoE: Ministry of Education
MoH: Ministry of Health
NEPI: Nursing Education Partnership Initiative
PEPFAR: (United States) President’s Emergency Plan for AIDS Relief
PMTCT: Prevention of mother-to-child transmission
PNLS: Programme National de Lutte Contre le VIH/SIDA, DRC’s national AIDS program
RR: Rural rotation (program for nursing students)
RRHS: Resilient and Responsive health systems
TB: Tuberculosis
WHO: World Health Organization
